# A one health approach to prioritizing emerging zoonotic diseases (EZDs) in northern Vietnam

**DOI:** 10.1016/j.onehlt.2025.101177

**Published:** 2025-08-21

**Authors:** Luong Hung Nam, Thang Nguyen-Tien, Johanna F. Lindahl, Sinh Dang-Xuan, Phuc Pham- Duc, Fred Unger, Bui Nghia Vuong, Dao Duy Tung, Hung Nguyen-Viet, Hu Suk Lee

**Affiliations:** a International Livestock Research Institute (ILRI), Viet Nam; b Department of Medical Biochemistry and Microbiology, Uppsala University, Sweden; c One Health laboratory, Division of Infectious Diseases, Department of Internal Medicine, University of Texas Medical Branch, USA; d Department of Animal Health and Antibiotic Strategies, Swedish Veterinary Agency, Uppsala, Sweden; e Center for Public Health and Ecosystem Research, Hanoi University of Public Health, Viet Nam; f Institute of Environmental Health and Sustainable Development, Viet Nam; g National Institute of Veterinary Research, Viet Nam; h International Livestock Research Institute (ILRI), Kenya; i College of Veterinary Medicine, Chungnam National University, Daejeon, Republic of Korea

**Keywords:** One health, Emerging zoonotic disease, Vietnam, Disease prioritization

## Abstract

**Background:**

Vietnam, with its significant agricultural sector, large livestock population, rich biodiversity and close human-animal interactions, is highly vulnerable to zoonotic disease transmission. To better address this threat, representatives from the human, animal, and environmental health sectors in Vietnam worked together at two one health zoonotic disease prioritization (OHZDP) workshops to develop a list of priority zoonotic diseases for multisectoral one health collaboration in Hoa Binh and Lao Cai provinces in April 2024.

**Methods:**

We modified the OHZDP process, developed by the U.S. Centers for Disease Control and Prevention (CDC), and utilized it to prioritize zoonotic diseases in Vietnam. This involved conducting defining prioritization criteria, developing specific questions with assigned weights for each criterion, and organizing two workshops with stakeholders.

**Result:**

There were 25 participants in Hoa Binh's workshop (7 females and 18 males), and 27 participants in Lao Cai's workshop (9 females and 18 males). Following a discussion of tool outputs among experts, four zoonotic diseases were prioritized in Lao Cai province: Rabies, avian influenza, *Streptococcus suis*, and leptospirosis. In Hoa Binh province, three diseases were identified as priorities: Rabies, avian influenza, and *Streptococcus suis.*

**Conclusion:**

Rabies, avian influenza and *Streptococcus suis* were the three most prioritized diseases across the two provinces. This list can serve as a foundation for strengthening one health collaboration for disease prevention and control in these targeted provinces.

## 1 Introduction

Emerging and re-emerging zoonotic diseases refer to those that have expanded or shifted their geographic, host, or vector range after their first recognition or identification [1]. Zoonosis are responsible for about 60 % of newly discovered infectious diseases, and they are common in domestic animals, poultry, livestock, and especially in wildlife species (71.8 %) [2]. The covid-19 pandemic, caused by the severe acute respiratory syndrome coronavirus 2 (SARS-CoV-2), serves as a stark reminder of the significant threat posed by zoonotic diseases. This global health crisis emphasizes the urgent need for proactive measures to prevent and control the emergence and spread of infectious diseases originating from animals [3]. Beyond emerging threats, endemic zoonosis contribute to an estimated 20 % of all human illnesses and deaths in the least developed countries and, in particular, people who live and work near livestock are at greater risk of contracting a zoonotic disease [4]. Globally, the 13 most impactful zoonotic diseases for poor livestock keepers in developing countries have been estimated to cause 2.7 million deaths and 2.4 billion human illness cases annually, with many of these diseases negatively affecting livestock production [5]. Among these diseases, rabies is the deadliest, causing approximately 70,000 human deaths annually [6].

Vietnam's unique socio-economic conditions, characterized by a predominantly rural population, intensive agriculture, and close human-animal interactions, have created a fertile ground for the emergence and spread of zoonotic diseases [2]. The country's geographical location in Southeast Asia, a region renowned for its rich biodiversity, facilitates the complex interplay between ecosystems, wildlife, and human health, which can increase the risk of zoonotic disease emergence.

The severe impact of emerging zoonotic diseases (EZDs) in Vietnam is evident in the historical outbreaks of highly pathogenic avian influenza (HPAI), which caused significant economic losses and public health concerns [[7], [8], [9]]. The emergence of other zoonotic threats, such as *Streptococcus suis* (*S.suis*) and leptospirosis, highlighted the ongoing challenge of managing these complex and evolving health risks [[10], [11], [12], [13], [14]].

A critical factor contributing to the vulnerability of Vietnam to zoonotic diseases is its limited capacity for surveillance and response. Organization-level barriers to implementing new practices include limited financial and personnel resources, difficulty accessing scientific literature, and the perceived applicability of research findings to rural settings [15]. Moreover, the vast number and diversity of zoonotic pathogens, each with their own unique epidemiological characteristics, pose significant challenges to effective management [16].

The One Health Systems Framework provides a structured approach to analyze and implement One Health initiatives by mapping interactions among human, animal, and environmental health, incorporating policy, institutions, stakeholders, and resources, with tools such as the One Health Zoonotic Disease Prioritization (OHZDP) supporting its application [17]. Therefore, to more effectively address challenges posed by EZDs in Vietnam, we conducted workshops which prioritized the EZDs of greatest national concern and also facilitated the development of EZD-specific multisector disease control and prevention strategies using the OHZDP tool.

## 2 Materials and methods

**Fig. 1:**
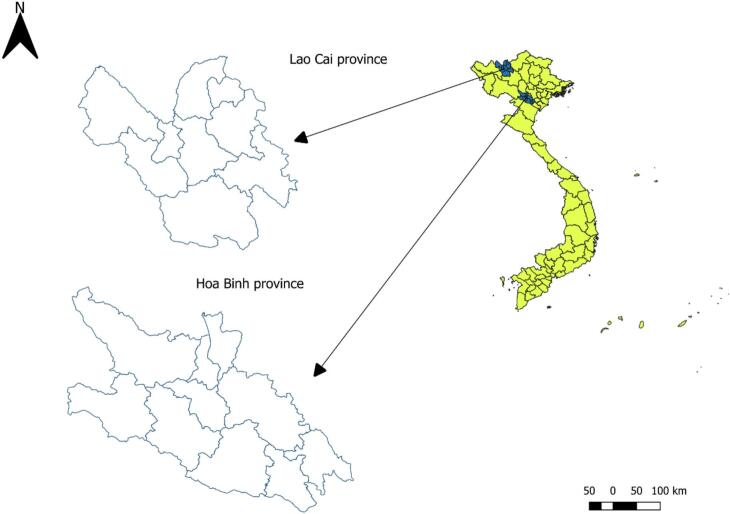
Selected two provinces were organized workshops (this figure was created by our team).

The consultation was held during two-day workshops in April 2024 in two provinces of Hoa Binh and Lao Cai (one workshop per province) in Northern Vietnam (Fig. 1). To select participants, government, research and academia institutions working on zoonotic diseases in the areas of surveillance, research and diagnostics in both human and animal health were identified. Then the invitations were sent requesting the nomination of experts on EZDs. To minimize systematic bias that could arise from participants from the same institutions sharing similar perspectives, participants from the same institutions were purposively assigned to different groups during group work sessions.

### 2.1 Prioritization tool and action groups

**Fig. 2:**
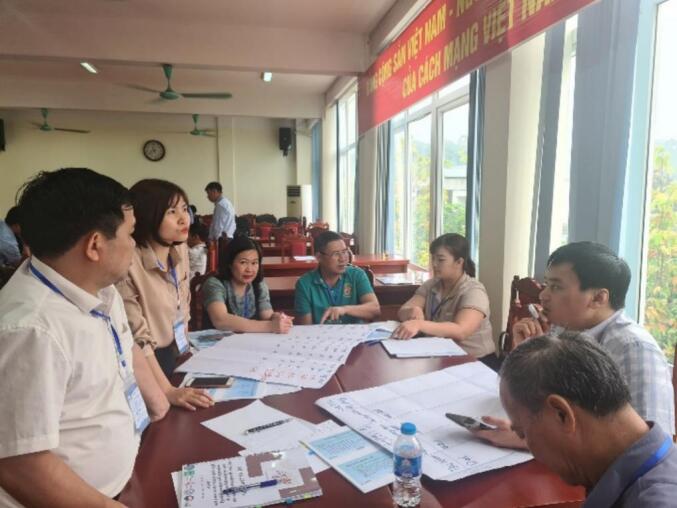
The action group consited of six to seven participants from CDC (who were standing), Sub-DAH (who were writing) and local veterinarians in workshop in Lao Cai province.

**Fig. 3:**
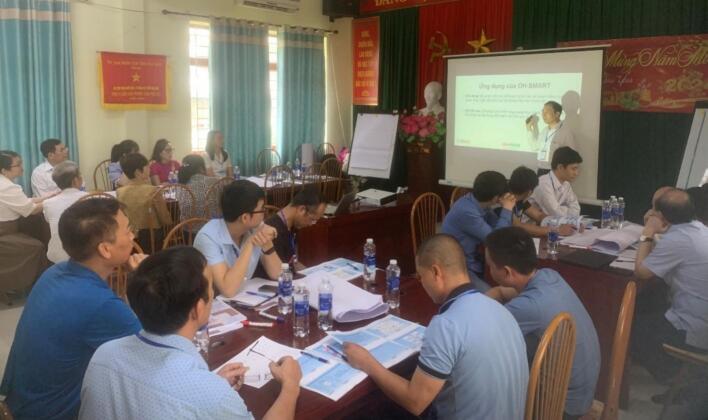
The action group consited of six to seven participants from CDC (who in the middle of picture), local health staff, Sub-DAH and local veterinarians were searching information in workshop in Hoa Binh province.

**Fig. 4:**
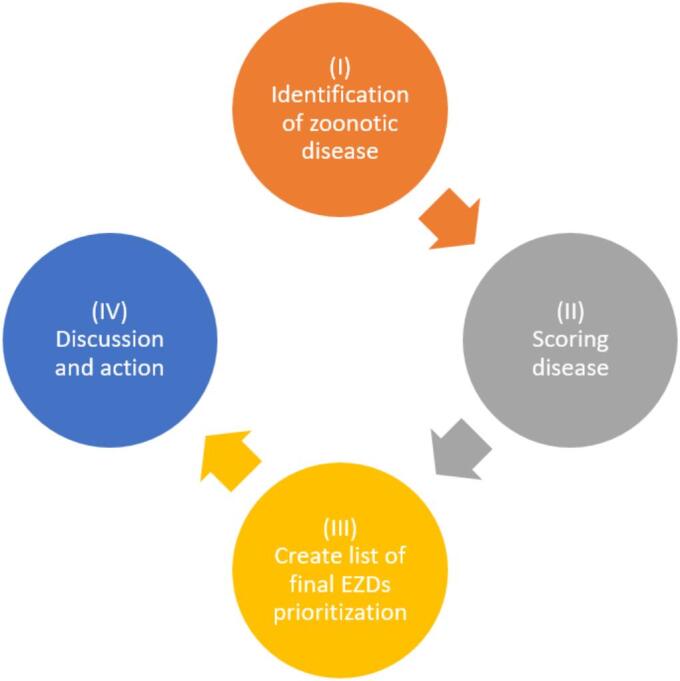
Emerging zoonotic diseases (EZDs) prioritization process during workshops.

We modified the OHZDP tool to allow the administration of action groups, where each group consisted of six to seven individuals, rather than the maximum of 12 participants as previously reported [18]. There was a total of six action groups from Lao Cai (3 groups) and Hoa Binh (3 groups) provinces. Each group comprised of one to two farmers, two to three public health and animal health professionals (working in surveillance, disease control, laboratory, academia, and research), and other relevant stakeholders from different sectors (e.g., environmental health officers, representatives from relevant departments and sub-departments of Food Hygiene and Safety; Culture, Sport and Tourism etc., agriculture centers) (Fig. 2**,** Fig. 3). The prioritization process consisted of four steps: (1) identifying zoonotic diseases to be prioritized, (2) scoring each disease based on the criteria, (3) voting by group members to create a final list of prioritized diseases, and (4) discussing actions to be taken (Fig. 4). The criteria and weighting scores used to create the list of zoonotic diseases are provided in Table 1. The final list of diseases to be prioritized was compiled by combining the lists of diseases/pathogens chosen by all six groups.

**Table 1:** Weighting for each criterion, and the categorical questions for each criterion and response options used to examine each of the EZDs selected for prioritization in targeted provinces.

Criteria (weight)	Question(s)	Responses and categories (score)
Severity of disease (0.4)	Is the disease serious in humans and animals? The severity of the disease is determined by the mortality rate. (In humans, one death is enough to consider the disease serious, while in animals, the disease is considered serious if the mortality or miscarriage rate is more than 5 %)	0: Not serious 1: Severe only in animals 2: Serious only in humans 3: Severe in both animals and humans
Epidemiological profile (incidence and prevalence) (0.22)	Have zoonotic diseases caused any outbreaks in humans in the past ten years?	0: No outbreak identified 1: Identified disease outbreak
Potential transmission (pandemic potentiality) (0.17)	Is the disease capable of being transmitted from one person to another? Person-to-person transmission includes all direct or indirect modes of transmission except interventional transmission (blood transfusion, needle stick, and organ transplantation).	0: When the disease has no reported cases of human-to-human transmission 1: When human-to-human transmission is found in “Some reported cases” 2: When cases of person-to-person transmission are continuously reported
Availability of intervention (0.13)	Are there any control and prevention measures for infectious diseases transmitted from animals to humans? This criterion evaluates the capabilities of local diagnostics, vaccination, surveillance, rapid response teams and risk communication measures.	0: When “No measures are available “ 1: When 1 or 2 measures are reported, as “Some measures available “ 2: When 3 to 4 measures are reported, “Most measures available “ 3: When “All available measures”
Social-economic- environmental impact (0.08)	How the disease affect production, trade and movement of animals and/or humans?	0: When no impact is recorded. 1: When the impact is recorded only in humans “Reduction in human labor productivity, impact on tourism and treatment and vaccination costs”. 2: When the impact is recorded only in animals “reduction in animal production and trade and treatment and vaccination costs” 3: When effects are noted in both animals and humans

### 2.2 Identification of zoonotic diseases to be prioritized

During the workshops, each group was required to create a list of zoonotic diseases relevant to their province, with a minimum of two diseases per group. The participants were allowed to search for publicly available data from websites such as the World Health Organization (WHO), the US CDC and other relevant resources from their respective provinces. To address data gaps, workshop participants supplemented the information with their knowledge, experience and consensus-based opinions. For a disease to be included on the list it had to be supported by case reports in Vietnam.

### 2.3 Applying the OHZDP tools: Criteria selection, disease scoring, and prioritization of zoonotic disease in Vietnam

The five criteria identified by previous research [19], included: severity of disease, epidemiological profile, potential transmission, availability of intervention, and social-economic-environmental impact, to rank EZDs include questions, responses, and categories for scoring according to Table 1. Each group's weighted criteria and corresponding question responses were applied to each zoonotic disease on their list to calculate a final disease score between 0 and 1. The final scores were calculated as the sum of the scores for each question. The scores for all diseases were then normalized relative to the highest scoring disease which was assigned a score of one. All participants in each group reviewed the disease ranking results, facilitating further discussion and consensus building. The final list of prioritized EZDs was created based on a list of all action groups. The diseases nominated by all three groups were included in the final list. The voting members then collaboratively finalized the list of priority zoonotic diseases for their province using these results.

The final approved list of diseases was subsequently used to inform the next steps and action plans to address threats related to zoonotic diseases of national significance. Our key performance indicators (KPIs) for One Health implementation include multisectoral coordination (e.g., inter-agency meetings), disease surveillance and response (e.g., detection-to-reporting time, jointly investigated outbreaks), capacity building (e.g., cross-sector trainings, laboratory capacity), and policy/governance (e.g., One Health–aligned policies, budget allocations) and regular biosecurity evaluation through Knowledge, attitude and practice (KAP) surveys to monitor and improve over time.

## 3 Results

The prioritization of EZDs was carried out through a facilitated consultative process involving 52 participants from different sectors within the One Health framework (Table 2**)**. These participants included representatives from human health (*n* = 11), animal health (*n* = 20), environmental health (n = 2), other related departments, sub-departments and agriculture centers (*n* = 7), as well as representatives of farmer households (*n* = 12) in Lao Cai and Hoa Binh provinces.

### 3.1 Hoa Binh province

There was a total of 25 participants (7 female and 18 male) including representatives from human health (*n* = 5), animal health (n = 11), environmental health (n = 1), the sub-department of food hygiene and safety (n = 1), the sub-department of culture, sport and tourism (n = 1) and farmer households (*n* = 6) in Table 2.

A list of diseases was created and scored by participants using prioritization criterion. Table 3 presents the raw and normalized score for zoonotic diseases in each group. In group 2, rabies was shown as the highest score (1.76), followed by *S. suis* (1.23) and avian influenza (0.82). While in group 3, rabies and *S. suis* had the same score (1.92), followed by covid-19 (1.83). In contrast to group 2 and 3, there were only two diseases listed: rabies (2.21) and liver fluke (1.01) in group 1.

**Table 3:** List of zoonotic diseases in Hoa Binh province.

#	Group 1			Group 2			Group 3		
	Disease	Raw score	Normalized final score	Disease	Raw score	Normalized final score	Disease	Raw score	Normalized final score
1	Rabies	2.21	1.00	Rabies	1.76	1.00	Rabies	1.92	1.00
2	Liver fluke	1.01	0.46	S.suis	1.23	0.70	*S. suis*	1.92	1.00
3				Avian influenza	0.82	0.47	Covid-19	1.83	0.95

After normalizing the final score of diseases, participants reached a final priority list that included three diseases (Table 4). Rabies was included since it was named by all three groups, whereas *S. suis* and avian influenza were chosen by all groups for the final EZDs list.

**Table 4:** Final zoonotic diseases selected in Hoa Binh province.

Rank	Zoonotic disease	Justification
1	Rabies	Same as the prioritized list
2	*S. suis*	Voting members agreement
3	Avian influenza	Voting members agreement

### 3.2 Lao Cai province

A total of 27 participants (9 female and 18 male) included representatives from human health (*n* = 7), animal health (*n* = 12), environmental health (n = 1), the sub-department of food hygiene and safety (n = 1) and representatives of farmer households (*n* = 6), presented in Table 2.

There were six diseases listed by participants: rabies, *S. suis,* avian influenza, covid-19, leptospirosis and liver flukes (Table 5). Avian influenza and rabies were listed by all three action groups and shared the top position on the list (rabies was ranked highest in group 4 and 6 while avian influenza was ranked highest in group 5). *S. suis* was also listed in two groups, but there was a score gap between the score of *S. suis* and the top-ranked disease (0.849 in group 4 and 0.723 in group 5).

**Table 5:** List of zoonotic diseases in Lao Cai province.

#	Group 4			Group 5			Group 6		
	Disease	Raw score	Normalized final score	Disease	Raw score	Normalized final score	Disease	Raw score	Normalized final score
1	Rabies	1.92	1.00	Avian influenza	1.84	1.00	Covid-19	1.83	1.00
2	*S. suis*	1.63	0.85	Rabies	1.76	0.96	Rabies	1.76	0.96
3	Avian influenza	0.82	0.43	Leptospirosis	1.71	0.93	Avian influenza	1.42	0.77
4				*S. suis*	1.33	0.72			

For the final list selected, rabies and avian influenza were chosen because of their high scores, whereas *S. suis* and leptospirosis received votes from 27/27 stakeholders (Table 6).

**Table 6:** Final zoonotic diseases selected in Lao Cai province.

Rank	Zoonotic disease	Justification
1	Rabies	Same as the prioritized list
2	Avian influenza	Same as the prioritized list
3	*S. suis*	Voting members agreement
4	Leptospirosis	Voting members agreement

### 3.3 Suggested next step

The following items were identified in workshops as crucial for disease control: (1) Proactive monitoring throughout the province, (2) Control of diseases in livestock, (3) Training for local officials, (4) Quarantine and slaughter control, and (5) Communication for farmers and the community as a common platform for reporting and sharing data on zoonotic diseases in humans and animals (Table 7). Participants identified the need to update preparedness and response plans that incorporate a One Health approach, as well as new plans for diseases that currently lack plans to improve capacity to respond to outbreaks.

**Table 7:** Suggested action to develop strategies against EZDs.

Solution	Proposed activities	Source	In charge
Proactive monitoring throughout the province	1. Develop an implementation plan	Local budget International project Domestic project	MARD, Sub-DAH
	2.Take monitoring samples		Local veterinary center
	3. Send test sample		Sub-DAH
	4. Analysis		NIVR
	5. Advise on intervention measures		Sub-DAH
Control diseases in livestock	1. Propagate and update diseases to livestock households	Local budget, Domestic/international project's budget	MOH, MARD, Sub-DAH
	2. Organize vaccination		
	3. Establish interdisciplinary rapid-response groups		
Training for local officials	1. Develop an implementation plan	International/ National project's budget	Sub-DAH, NIVR
	Design appropriate lessons and methods according to local wishes		NIVR
	Choose time, location and activity budget		Sub-DAH, NIVR
Quarantine and slaughter control	1. Quarantine/control of imported animals and animal products	Government budget	Customs authority Sub-DAH
	2. Quarantine of centralized slaughterhouses		
Communication between farmers and the community	1. Developing Communication materials (flyers, posters)	International/ national projects	Sub-DAH
	2. Develop a direct/indirect communication plan		

## 4 Discussion

From our findings, the list of zoonotic diseases showed some commonalities and differences when compared with results of other zoonotic disease prioritization workshops. Out of the top five priority zoonotic diseases in neighboring China (avian influenza, echinococcosis, rabies, plague, and brucellosis), only two of them were included in this study's list of priority zoonotic diseases: avian influenza and rabies [20]. However, in contrast to China, Vietnam chose rabies as its most prioritized zoonotic disease in five out of the six groups, while rabies was within the top three in China's list. From 2005 to 2015, Vietnam reported 4,196,247 (average 429.55 per 100,000) rabies post-exposure prophylaxis (PEP) cases and 934 fatalities (average 0.10 per 100,000) [21]. Within nine years from 2005 to 2014, rabies cost Vietnam approximately USD 719 million, with PEP accounting for 92 % of the total cost [22]. During this time, rabies led to 36,560–45,700 disability-adjusted life years in the population. The Prime Minister of Vietnam has issued a national action plan for rabies control covering the period from 2022 to 2030, based on the One Health approach [23]. This plan aims to control rabies in the animal sector throughout this period, with specific targets for 2022–2025 and 2026–2030. Specific objectives include managing 70 % and 90 % of the dog and cat population, respectively, vaccinating 70 % of dogs and 80 % of cats, and establishing 10 rabies-free zones at the district and commune levels. These plans were also mentioned by the Sub-Department of Animal Health (DAH) staff in our workshops. The significant economic burden associated with rabies, including the loss of livestock and the costs associated with PEP, further show the importance of prioritizing this disease – there is a reason why people selected rabies as the most important disease in their provinces. Avian influenza was prioritized in both China and Lao Cai province, Vietnam, which shares the border with China. A previous study conducted in Quang Ninh province, adjacent to China, identified four key risk factors for avian influenza outbreaks: raising poultry in uncovered or partially covered ponds, visits from poultry traders, farms with 50–2000 birds, and farms with ≥2000 birds [24]. During the workshop, in addition to the proximity of farms to the shared border with China, local veterinarians further commented that small and medium scale farmers lack sufficient knowledge and experience in biosecurity. Workshop participants agreed that avian influenza outbreaks can have devastating consequences for the poultry industry, leading to mass culling of infected birds, significant economic losses for farmers, and disruptions to the food supply chain. Many provinces in Vietnam, including Lao Cai and Hoa Binh provinces, have significant poultry industries, and outbreaks can have a major impact on the livelihoods of many people. These factors likely contributed to the participants' decision to prioritize avian influenza as a major zoonotic disease threat in their provinces. Avian influenza and rabies were also mentioned in the list of prioritized diseases in India – another agricultural country in Asia [25]. Similar to the current study, rabies was evaluated as the most prioritized disease in India. Comparing our finding to other countries in Asia [20,25], all six diseases in our list were also mentioned in the lists of other countries but with different rankings.

Rabies and Avian Influenza (AI) are compelling examples of the One Health concept, illustrating the intricate links between animal, human, and environmental health [26,27]. From 2016 to 2021, the regional rabies mortality rate was 0.41 deaths per 100,000 people, a rate that remains high and contrasts with national elimination goals. This high rate, with fatalities concentrated in provinces such as Phu Yen and Binh Thuan in Vietnam, emphasizing the urgent need for targeted, integrated One Health interventions [28]. Overall, rabies-related fatalities were recorded sporadically across eight of the region's 11 provinces, with a concerning concentration of deaths in Phu Yen, Binh Thuan, Binh Dinh, Quang Nam, and Quang Ngai [29]. Their spread is strongly influenced by environmental conditions, including wild bird migration patterns and on-farm biosecurity practices. The control of both diseases necessitates a holistic, multisectoral strategy that integrates expertise from public health, veterinary medicine, and environmental management to be effective. From 2003 to 2014, Vietnam reported 127 human cases of H5N1 highly pathogenic avian influenza (HPAI), resulting in 63 fatalities [12]. In 2020, outbreaks of HPAI were notified in 84 communes across 28 provinces, leading to the culling of 255,209 poultry.

As mentioned above, by focusing on individual pathogens rather than diseases, this approach allowed us to conduct an unbiased prioritization, avoiding potential biases stemming from programmatic considerations. This enabled us to compare the relative importance of pathogens within and across different disease groups. For example, we were able to identify differences in the prioritization of leptospirosis and *S. suis*, even though both are transmitted infections from pigs. *S. suis* was known by both experts and livestock farmers while information about leptospirosis was still limited. *S.suis* in swine was documented to be as high as 41 % in some others studies in Southeast Asia [[30], [31], [32]] and was remarkably low at industrial swine farms in Northern Vietnam based on the studies in 2021 [11]. These findings may help to explain why large farms are better equipped to manage and control the spread of disease through biosecurity measures. Other studies have demonstrated that staff members at small-scale swine farms adhere to personal protective equipment (PPE) at a low rate and that biosecurity protocols are less strict [11]. In the workshop, local staff in Sub-DAH also commented that there were difficulties in addressing the awareness and behavior of farmers, in their farm activities, and that there were barriers in sharing knowledge with farmers. The variations in regulatory adherence are most likely attributable to inequalities in resource availability, since small-scale farms have less discretionary cash to invest in disease control and prevention. Given that backyard and small-scale pig farming operations account for a substantial amount of Vietnam's pork supply chain, more studies will be required to identify challenges to proper sanitation and PPE adherence in these contexts.

*Streptococcus suis* highlights the interconnectedness of human and animal health within the agricultural context. It is a significant pathogen in pigs, causing a range of diseases. The bacterium can be transmitted to humans through direct contact with infected pigs or through the handling of contaminated pork products, leading to severe illness. In domestic pigs in Vietnam, the infection rate fluctuated significantly, ranging from 0 % to 85.19 % between 2011 and 2019 [12]. From 2011 to 2018, there were between 55 and 173 annual hospitalizations due to *S. suis* type 2 in humans in Vietnam [12]. The morbidity rate peaked in 2017 at 0.19 cases per 100,000. In domestic pig populations, the infection rate fluctuated significantly, ranging from 0 % to 85.19 % between 2011 and 2019. The prevalence of this disease is intricately linked to environmental factors, including biosecurity and sanitation on pig farms, as well as effective waste management. One Health approach is therefore essential for addressing this pathogen by focusing on improving farming practices to protect both animal and human populations.

Leptospirosis is a classic One Health issue, demonstrating the interconnections between animals, humans, and the environment [33]. Humans can become infected through direct contact with infected animals or, more often by exposure to contaminated environments, such as floodwater, soil, or food and water sources [34]. There were 85 human cases officially reported in Vietnam between 2008 and 2015 [35]. A previous study in 2023 showed that *Leptospira* seroprevalence among healthy Vietnamese individuals was 9.5 %, with farmers making up the majority of these cases (63.2 %) [13]. However, the actual prevalence is likely higher due to underreporting, particularly in rural areas where public awareness and diagnostic facilities are limited. To address this, it is crucial to raise awareness among high-risk groups such as farmers, miners, veterinarians, and slaughterhouse workers, who are more susceptible to exposure to infected animals and contaminated environments. Leptospirosis presents a significant occupational hazard for individuals working in agriculture, mining, sewer maintenance, veterinary medicine, and other professions that involve exposure to contaminated water, soil, or infected animals [36]. In pigs, a previous study conducted in 2019 across ten provinces in Vietnam revealed a seroprevalence of leptospirosis of 21.05 % [35]. It is important to consult veterinarians and animal health experts for specific advice on leptospirosis prevention and control in pigs. Besides, other domestic animals can also be infected with leptospirosis. A previous study conducted in 2021, using the microscopic agglutination test (MAT), found high seroprevalence rates of *Leptospira* in various animal species in Vietnam [37]. Specifically, 44.2 % of buffaloes, 24.9 % of cattle, 10.2 % of swine, 32.9 % of dogs, 12.2 % of cats, and 16 % of rats tested seropositive for *Leptospira*. These findings highlighted the widespread circulation of *Leptospira* among animals that have been in close contact with humans, emphasizing their potential role as significant sources of human leptospirosis transmission in Vietnam.

The One Health approach integrates public health, veterinary, and environmental sectors to implement coordinated interventions (e.g., vaccination campaigns), enhance cross-sector communication for rapid outbreak response, and inform evidence-based policy and research through a comprehensive understanding of disease ecology.

Zoonotic illnesses have significant economic and public health impacts, highlighting the significance of coordination between the human and animal health sectors as well as the necessity of surveillance, prevention, and control [38]. This report from WHO also showed that long-standing deficiencies in addressing emerging diseases, such as (a) readiness and quick response, (b) public health infrastructure, (c) efficient and fast risk communication, (d) research, and (e) political commitment, as well as national and international collaborations, have been brought to light by the covid-19 pandemic [38].

The covid-19 pandemic highlighted the urgent need for such a plan, emphasizing the importance of effective measures to prevent and combat zoonotic diseases. Vietnam's prioritization process serves as a model for the region, demonstrating the value of a multi-sectoral approach. The One Health approach was central to the workshops, fostering collaboration among diverse stakeholders and ensuring that all voices, including those of livestock farmers, were heard. This inclusive approach facilitated the identification of knowledge gaps and the development of a more comprehensive plan for addressing zoonotic diseases in Vietnam.

Although the study adhered to the fundamental tenets of the CDC's OHZDP tool, specific adaptations were implemented to enhance its applicability within the Vietnamese context. Firstly, the standard methodology of a single large group was replaced with three smaller, facilitated groups, each comprising six to seven participants. This modification addressed the potential for power imbalances within a larger group, particularly given the inclusion of farmers alongside animal and human health experts. The small group format fostered a more inclusive environment, ensuring all participants, regardless of professional background, were able to contribute their experiences, narratives, and justifications for disease scoring. This approach served as the primary method for data collection from participants. Secondly, the initial zoonotic disease priority list was generated collaboratively by the workshop participants, rather than utilizing a pre-existing list derived from official governmental publications or peer-reviewed literature, as commonly employed in previous international studies [19,39]. This approach was adopted to capture real-time epidemiological information directly from provincial stakeholders. While the number of diseases identified per group was constrained (minimum two diseases), this methodology yielded a more accurate reflection of the current disease landscape within the participating provinces. These modifications were intentionally implemented to maximize the tool's relevance and applicability to the local context. Nevertheless, it is acknowledged that these adaptations may have influenced the final disease prioritization outcomes.

The workshop outcomes included a prioritized list of zoonotic diseases and recommendations for multisectoral engagement in disease control and prevention. It recognized the need for improved intersectoral coordination, particularly between the Ministry of Agriculture and Rural Development (MARD) and the Ministry of Health (MOH), along with the Sub-Department of Animal Health (Sub-DAH) and local veterinary centers. To address this, the workshop advocated for strengthening Vietnam's One Health workforce through regular collaboration training, simulation exercises, and other preparedness and response education opportunities. Drawing inspiration from pandemic influenza exercises conducted by the China CDC [40,41], the participants emphasized the value of expanding these exercises to encompass other sectors and address priority zoonotic diseases in Vietnam.

Although this workshop was organized after the covid-19 pandemic, highlighting its effectiveness in regional and Vietnamese contexts, covid-19 was not prioritized in the final list while other diseases such as rabies, avian influenza, and *S. suis* were chosen instead. However, after the initial spillover, the role of animals in covid-19 transmission was minimal, and therefore the participants probably did not consider it as a zoonosis in the exercise [42]. Another factor that may have influenced the results is the lack of comprehensive national-level data on zoonotic diseases, particularly in MARD where data is often recorded annually, and detailed monthly or case-specific records are missing. This data limitation may have skewed prioritization towards MOH's perspective, which has access to more up-to-date data. However, efforts were made to mitigate this bias by involving experts from Sub-DAH, local veterinarians, and Vietnam CDC to consider regional and global data. Despite these efforts, the limitations in data availability highlighted the need for enhanced surveillance and data sharing. While there may be varying perspectives on the validity of the prioritization, the exercise's significance lies in its transparency and the relative ranking of diseases compared to one another, regardless of the specific methodology employed [16,43].

Another limitation of this study is its geographic scope. Focusing on only two provinces, Hoa Binh and Lao Cai in Northern Vietnam, may limit the generalizability of the findings to the entire country. Vietnam has diverse ecological and socio-economic conditions, and zoonotic disease priorities may vary significantly across different regions [44]. In addition, limitations of zoonotic disease reporting, due to factors such as lack of awareness, surveillance systems, and limited diagnostic capacity, can lead to the underestimation of the true burden and impact of disease. While the methodology and findings of this study may offer valuable insights for other regions, it is important to acknowledge that the specific priorities identified may not be directly transferable to areas with different ecological, socio-economic, and epidemiological contexts. These limitations should be acknowledged and considered when interpreting the findings of this research and developing future One Health interventions in Vietnam.

## 5 Conclusion

By employing the OHZDP tool, a prioritized list of zoonotic diseases was established in Vietnam, laying the groundwork for a One Health approach. Key priorities identified include rabies, avian influenza, and *Streptococcus suis*. Rabies, recognized as a significant public health burden with substantial economic impacts, emerged as the top priority in five out of six groups, reflecting its critical importance in Vietnam. Avian influenza, a significant threat to both human and animal health, was prioritized due to its potential for rapid spread and severe economic impacts on the poultry industry. Similarly, *Streptococcus suis*, a significant zoonotic pathogen primarily affecting pigs, was prioritized due to its impact on both animal health and the potential for severe human infections, particularly among those with occupational exposure to swine.

The One Health approach has facilitated improved multi-sectoral planning, communication, and collaboration among human, animal, and environmental health sectors. To realize the full potential of the One Health approach in Vietnam, targeted capacity-building and training programs should be implemented across all provinces. These programs should focus on equipping stakeholders with the necessary skills and knowledge to effectively implement the OHZDP tool, conduct multi-sectoral risk assessments, and develop integrated disease control strategies. By strengthening local capacity, Vietnam can build a sustainable One Health infrastructure that protects the health of people, animals, and the environment.

## Author statement

Luong Hung Nam: Writing – original draft, data collection, data analysis, visualization, and interpretation of data. Thang Nguyen-Tien: data collection, review, and editing. Johanna F Lindahl: review, and editing. Sinh Dang-Xuan: data collection, review, and editing. Phuc Pham- Duc: data collection, review and editing. Fred Unger: review and editing. Bui Nghia Vuong: review and editing. Dao Duy Tung: review and editing. Hung Nguyen-Viet: review and editing. Hu Suk Lee: supervision, conceptualization, methodology, data analysis, interpretation of data, review, and editing.

All authors read and approved the final manuscript.

## Funding

This work was funded by the 10.13039/501100003624Ministry of Agriculture, Food and Rural Affairs (MAFRA), Republic of Korea. In addition, this work was supported by the 10.13039/501100003725National Research Foundation of Korea (NRF) grant funded by the Korean government (MIST) (No. RS-2021-NR060136) and 10.13039/501100014189Korea Institute of Planning and Evaluation for Technology in Food, Agriculture and Forestry (IPET) through High-Risk Animal Infectious Disease Control Technology Development Project, funded by 10.13039/501100003624Ministry of Agriculture, Food and Rural Affairs (MAFRA).

## Declaration of competing interest

We declare no competing interests.

## Figures and Tables

**Table:** 

Time	Contents	Person in charge
8:00–8:30	Registration and Welcome	NIVR
8:30–9:00	Introduction to One Health Approach and Workshop Objectives	IEHSD/VOHUN experts
9:00–10:00	Overview of Zoonotic Diseases in Global, Vietnam, Hoa Binh	IEHSD/VOHUN experts
10:00–10:15	Coffee Break	All
10:15–12:00	Group Discussions: Prioritizing Zoonotic Diseases: • Participants divided into small groups representing different sectors (4 groups) • Each group discusses and prioritizes zoonotic diseases based on predefined criteria (e.g., severity, prevalence, economic impact, feasibility of control measures) • Facilitators guide discussions and ensure representation from all sectors.	Facilitators: IEHSD/VOHUN experts All participants
12:00–13:30	Lunch Break	All
13:30–14:30	Each group reporting & consensus. • Representatives from each group present their prioritized list of zoonotic diseases. • Facilitated discussion to reconcile differences and reach consensus on the final list. • Agreement on top priority zoonotic diseases for Hoa Binh province	Facilitators: IEHSD/VOHUN experts All
14:30–15:30	Action Planning • Identification of key actions and interventions required to address the prioritized zoonotic diseases. • Assignment of responsibilities to relevant stakeholders. • Discussion on resource mobilization and collaboration mechanisms	Facilitators: IEHSD/VOHUN experts All
3:30–3:45	Coffee Break	All
3:45–4:45	Open discussion and next steps: • Summary of key outcomes and decisions from the workshop • Discussion on follow-up activities and monitoring mechanisms • Expression of gratitude to participants and facilitators	Facilitators: IEHSD/VOHUN experts All
4:45–5:00	Workshop Conclusion	NIVR

**Table 2:** List of stakeholders who have participated in the zoonotic disease prioritization in Lao Cai and Hoa Binh province during participatory workshops.

Department	Sector	Number of participants	
		Female	Male
Hoa Binh		7	18
Agriculture service (province)	Animal Health	1	1
Sub-DAH	Animal Health	1	5
Local veterinarian (District/ Commune vet)	Animal Health	1	2
Health Service (province)	Human Health	0	1
CDC	Human Health	0	2
Health service (district/ commune)	Human Health	1	1
Sub- Department of Environment	Environmental Health	1	0
Sub-Department of Food Hygiene and Safety	Others	0	1
Sub-Department of Culture, Sport and Tourism	Others	0	1
Livestock farmers	Others	2	4
Lao Cai		9	18
Agriculture service (province)	Animal Health	0	1
Sub-DAH	Animal Health	1	2
Local veterinarian (District/ Commune vet)	Animal Health	1	7
Health Service (province)	Human Health	0	1
CDC	Human Health	1	0
Health service (district/ commune)	Human Health	2	3
Sub- Department of Environment	Environmental Health	0	1
Sub-Department of Food Hygiene and Safety	Others	0	1
Livestock farmers	Others	4	2

## Data Availability

Data will be made available on request.
